# Supplementary figures and images for: Notch signaling regulates vasculogenic mimicry and promotes cell morphogenesis and the epithelial-to-mesenchymal transition in pancreatic ductal adenocarcinoma
Source: PLoS One. 2022 Dec 22;17(12):e0279001. doi: 10.1371/journal.pone.0279001 (PMC9779037; doi:10.1371/journal.pone.0279001)

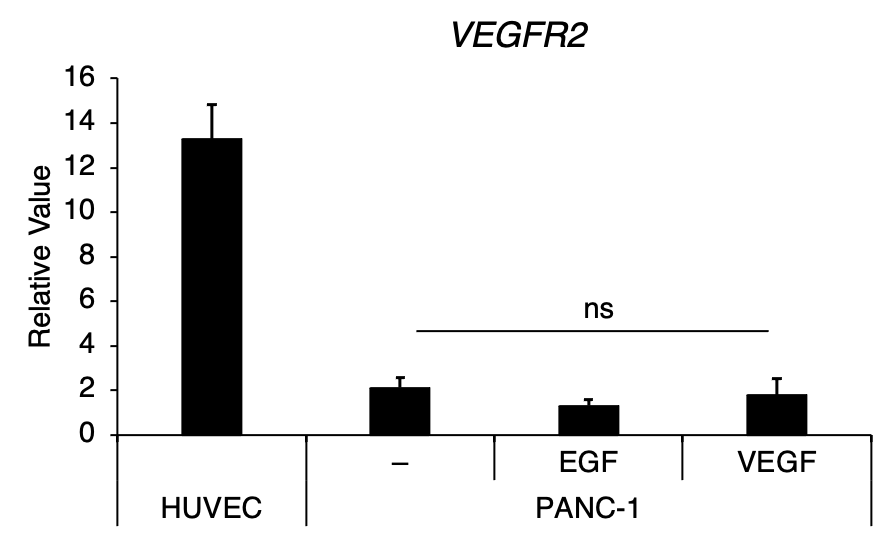

Supplement: S1 Fig — HUVEC and PANC-1 were treated with 20 ng/ml of either EGF or VEGF and cultured for 48 hours. The expression of VEGFR2 was determined by quantitative RT-PCR. Data presented ± S.D. (n = 3). (TIF) [file pone.0279001.s001.tif]

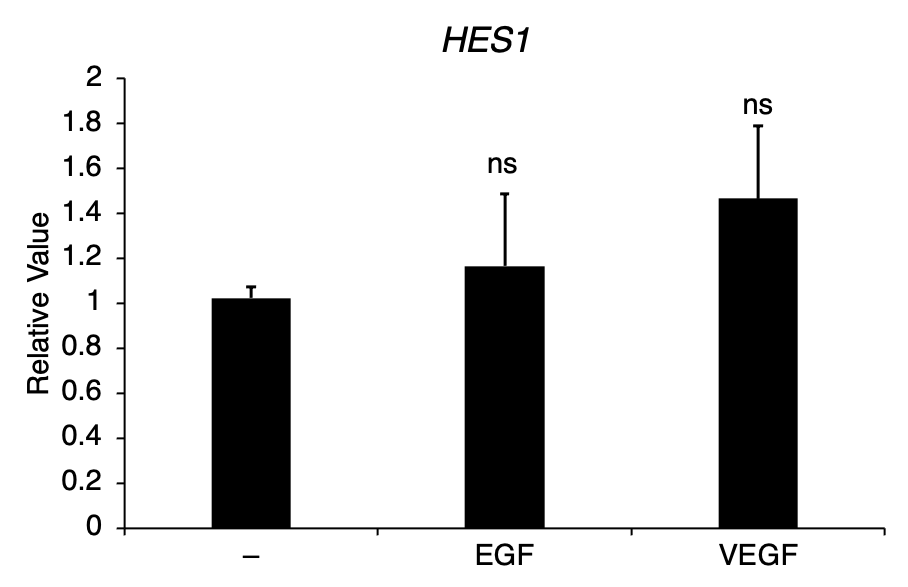

Supplement: S2 Fig — PANC-1 cells were treated with 20 ng/ml of either EGF or VEGF and cultured for 48 hours. The expression of HES1 was determined by quantitative RT-PCR. Data presented ± S.D. (n = 3). (TIF) [file pone.0279001.s002.tif]

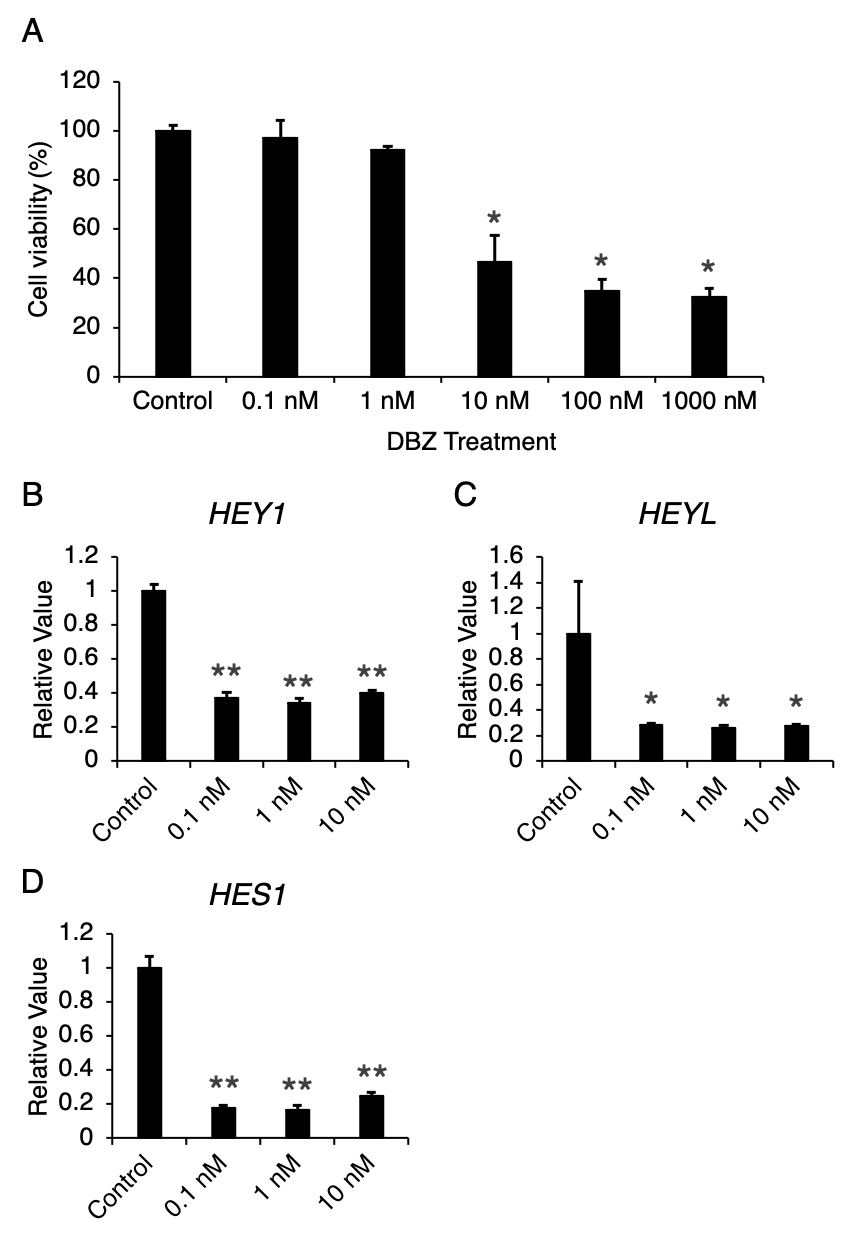

Supplement: S3 Fig — (A) PANC-1 cells were treated with 0.1 nM, 1 nM, 10 nM, 100 nM, and 1,000 nM DBZ and cultured for 48 hours. Cell viability was not affected at 0.1 and 1 nM DBZ but significantly decreased at higher concentrations. Data presented ± S.D. * P Value < 0.001 (n = 3). (B-D) The expression of HEY1, HEYL, and HES1 was determined by quantitative RT-PCR. Data presented ± S.D. *P Value < 0.01, **P Value < 0.001 (n = 3). (TIF) [file pone.0279001.s003.tif]

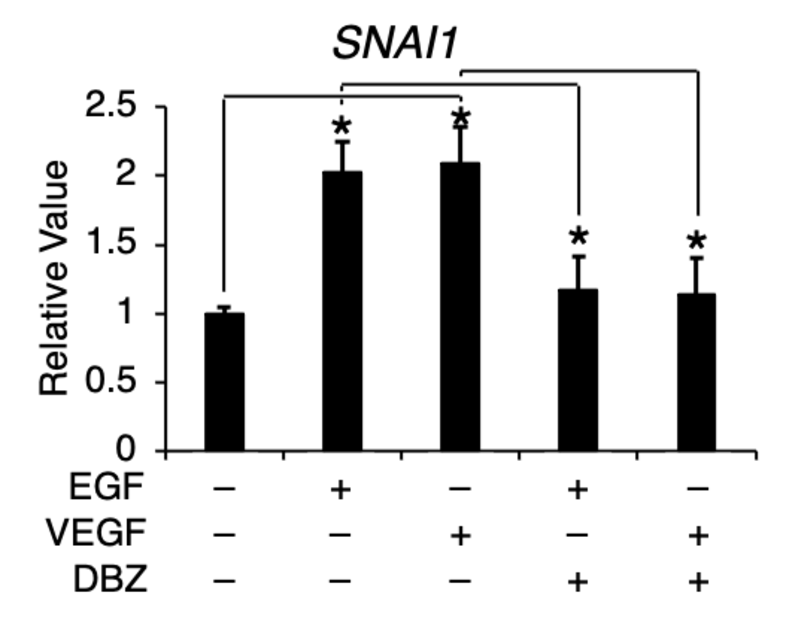

Supplement: S4 Fig — PANC-1 cells were treated with 20 ng/ml of either EGF or VEGF or 1 nM DBZ and cultured for 48 hours. The expression of SNAI1 was determined by quantitative RT-PCR. Data presented ± S.D. * P Value < 0.01 (n = 3). (TIF) [file pone.0279001.s004.tif]

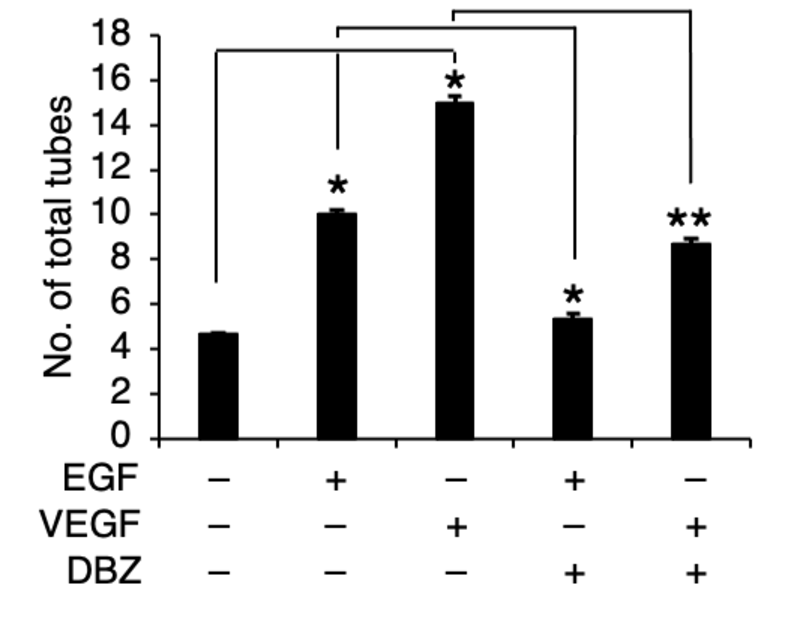

Supplement: S5 Fig — PANC-1 cells were seeded in Matrigel and treated with 20 ng/ml of either EGF or VEGF and cultured for 24 hours. Network formation was quantitated by Wimasis Image Analysis. Data presented mean number of total tubes ± S.D. (n = 3). (TIF) [file pone.0279001.s005.tif]

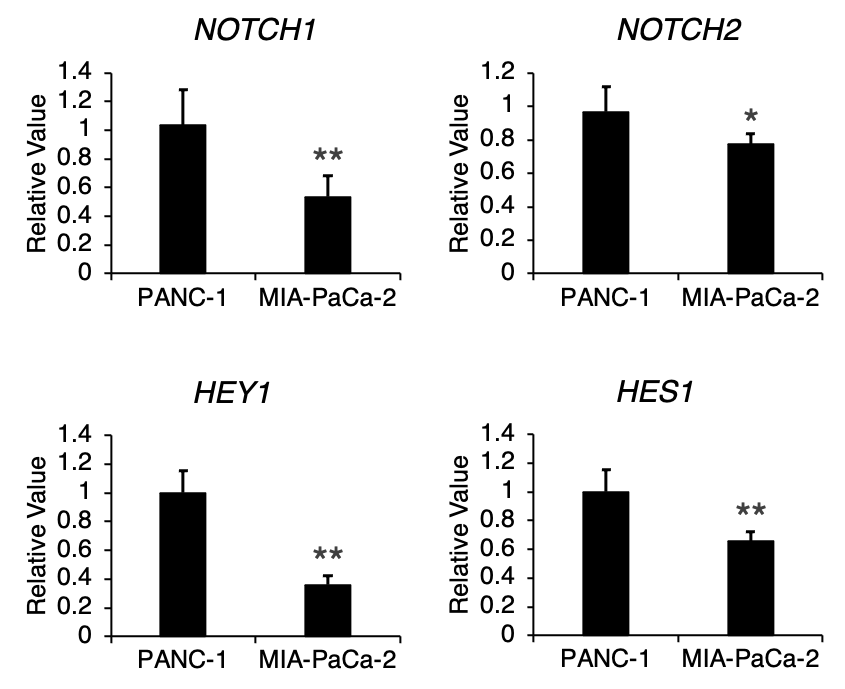

Supplement: S6 Fig — Both cells were cultured up to 80–90% confluency before analysis. Gene expression was determined by quantitative RT-PCR. Data presented ± S.D. *P Value < 0.05, ** P Value < 0.01 (n = 3). (TIF) [file pone.0279001.s006.tif]
